# Transcriptomic and Metabolomic Data Reveal the Key Metabolic Pathways Affecting *Streltzoviella insularis* (Staudinger) (Lepidoptera: Cossidae) Larvae During Overwintering

**DOI:** 10.3389/fphys.2021.655059

**Published:** 2021-06-18

**Authors:** Jiahe Pei, Yabei Xu, Shixiang Zong, Lili Ren

**Affiliations:** Beijing Key Laboratory for Forest Pest Control, Beijing Forestry University, Beijing, China

**Keywords:** *Streltzoviella insularis*, overwintering, transcriptome, metabolome, metabolic pathway

## Abstract

*Streltzoviella insularis* (Staudinger) (Lepidoptera: Cossidae) is a woodboring insect feeding on *Fraxinus pennsylvanica*, *Sophora japonica*, and *Ginkgo biloba*, as well as many other species used for urban greening and plain afforestation in northern China, including the temperate north. There is also a risk that *S. insularis* could spread through the transportation of seedlings, thereby increasing urban greening costs. However, how *S. insularis* increases the cold tolerance then reduces it to survive winter temperature below 0°C remains unclear. In the transcriptomic of *S. insularis*, we identified three profiles (profile 25, 27, and 13) whose trends related to the cold tolerance. We detected 1,783 differentially expressed genes (in profile 25) and identified 522 genes enriched in the AMPK signaling pathway. The metabolome analysis identified 122 differential metabolites. We identified four co-pathways, among which “Glycerophospholipid metabolism” was the pathway most enriched in differentially expressed genes and differential metabolites. The AMPK signaling and glycerophospholipid metabolism pathways play key roles in the natural overwintering physiological process of *S. insularis* larvae.

## Introduction

*Streltzoviella insularis* (Staudinger) (Lepidoptera: Cossidae) is a woodboring insect feeding on *Fraxinus pennsylvanica*, *Sophora japonica*, *Ginkgo biloba*, *Broussonetia papyrifel* (Gao and Qin, 1983), as well as many other species used for urban greening and plain afforestation in northern China, even including the temperate north. We also observed that, compared with *F. bungeana* DC, *S. insularis* caused greater harm to *F. pennsylvanica* Marsh. var. subintegerrima (Vahl), which was similar to *Agrilus planipennis* Fairmair (Zhao et al., 2007). There is also a risk that *S. insularis* could spread through the transportation of seedlings, thereby increasing urban greening costs.

Insects are poikilotherms and deploy cold tolerance as their principal survival strategy in low-temperature environments. Insects have been reported to use supercooling to enhance cold tolerance, thus avoiding the freezing of body fluids at temperatures below 0°C. Common cold-tolerant strategies for insects include freeze tolerant, freeze avoidant, and chill susceptible (Sinclair, 1987, 1993, 1996, 1999; Sinclair et al., 2015). In our previous study on the cold tolerance of *S. insularis* larvae, we identified freeze tolerance as the likely strategy; that is, when their body temperature is higher than the lethal temperature but below 0°C, body fluids freeze but keep the intracellular fluids from freezing, so that they can survive. Also, the larvae of *S. insularis* increased the ability of cold tolerance first then reduced during overwintering and showed the strongest cold tolerance at the lowest point of environmental temperature, and there was no diapause (Pei et al., 2020). However, as the temperature first decreased and then increased, why cold tolerance of *S. insularis* larvae first increased then decreased remains unclear.

When insects exhibit cold tolerance during the overwintering period, their metabolism changes from anabolic (feeding, growth, and storage of nutrients) in summer and early autumn to catabolic (synthesis of antifreeze agents, consuming stored energy) (Storey and Storey, 2012). This transition is triggered by the lowering of external temperature in late autumn and winter, so the metabolism changes involve multiple signaling pathways. Simultaneously, the types and contents of metabolites in insects during the overwintering period will also change. High concentrations of low molecular weight sugars and alcohols, such as glycerol and sorbitol, are often used as antifreeze agents by insects (Storey and Storey, 1988; Sømme, 1964, 1965; Feng et al., 2016; Park et al., 2017; Burns et al., 2020). Trehalose is also used as an important component of antifreeze agents in many insects and helps to stabilize the lipid bilayer of the membrane, especially during the cell volume shrinking in freeze-resistant insects, whose content will vary significantly (Koštál et al., 2011; Feng et al., 2016; Park et al., 2017; Burns et al., 2020). Our previous studies have shown that the total lipid content and total glycogen content of *S. insularis* larvae were closely related to changes in cold hardiness (Pei et al., 2020). Glycerophospholipids are the main membrane lipid constituents (Pilkis and Granner, 1992). Research on *Pyrrhocoris apterus* shows that it changes the composition of glycerophospholipids in the body under low-temperature stimulation (Tomcala et al., 2006). Studies on cold shock of fruit fly (*Drosophila melanogaster*) had shown that changes in cell membranes were more likely to occur during long term cold stimulation (Overgaard et al., 2008; Koštál et al., 2011).

Multi-omics interactive analysis is a method that uses high-throughput omics methods to describe biological processes, including genomics, transcriptomics, proteomics, and metabolomics. Through the integrative analysis of data, a comprehensive understanding of systems biology can be achieved. The transcriptomics and metabolomics can explain changes in organisms from the level of gene transcription and small molecule metabolites. The interactive analysis of transcriptomics and metabolomics is based on the bridge of metabolic pathways, which can explain the metabolic changes in organisms (Cavill et al., 2016; Misra et al., 2019).

Transcriptomic and metabolomic studies on the cold tolerance of *Drosophila melanogaster* have shown that proline and glutathione metabolism play important roles in the process of cold tolerance (MacMillan et al., 2016). Transcriptomic and metabolomic studies have shown that glycolysis, gluconeogenesis, and the tricarboxylic acid cycle (TCA cycle) play important roles in the process of cold tolerance of *Corythucha ciliate* (Li et al., 2017). The interactive omics research on *Phanaeus vindex* showed that acclimation to different temperature fluctuations is distinct and may be supported by increasing transcriptional plasticity (Sheldon et al., 2020). However, to our knowledge, the molecular mechanism of forest borers’ cold hardiness has not been addressed using an interactive analysis of the omics approach.

This study investigated the changes in transcription and metabolism levels of the larvae of *S. insularis* throughout the overwintering period (October 2018–March 2019), aiming to explore the key metabolic pathways that affect their cold tolerance under natural conditions.

## Materials and Methods

### Insects Collecting

*S. insularis* larvae with their living wood sections were collected from street trees (*F. pennsylvanica*) at south Xiangshan Road, Shijingshan District, Beijing, China (N 39° 57′, E 116° 12′). The wood sections with larvae were stored in open areas where the temperature can be monitored, and the temperature was recorded once a month using a thermometer (L91-1, Hangzhou loggertech Co., Ltd, China) (Pei et al., 2020). The monthly temperature records in Beijing are also shown in Supplementary Figure 1. From September 2018 to March 2019, 11 larvae were collected every 2 weeks, quickly frozen in liquid nitrogen, and stored in a refrigerator at −80°C. Per 11-larvae sampling, three larvae were used for transcriptome sequencing (three biological replicates), five larvae were for metabolome determination (five biological replicates), and three larvae were used for qRT-PCR to clarify the reliability and universality of the transcriptome (three biological replicates). According to the changing trend of the supercooling point, 1 month after the 12th sampling (numbered SI_1∼12), the 13th sampling was used as the control group (numbered SI_13). At this time, the larvae had completed their overwintering (Pei et al., 2020).

### Total RNA Extraction

Total RNA was extracted from larvae using RNeasy Plus Mini Kit (No. 74134; Qiagen, Hilden, Germany) following the manufacturer’s instructions. RNA purity, concentration, and integrity were assessed using NanoDrop2000 (Thermo, Waltham, MA, United States) and Agarose gel electrophoresis.

### cDNA Library Construction and Transcriptome Sequencing

Sequencing libraries were constructed using the Illumina Truseq^TM^ RNA sample prep kit following standard procedures. cDNA libraries were sequenced on the Illumina HiSeq 4000 platform at Majorbio Bio-pharm Technology Co., Ltd. (Shanghai, China). The software fastx_toolkit_0.0.14^1^ was used to perform a quality evaluation of the original sequencing data of each sample, base quality distribution statistics, base error rate distribution statistics, and A/T/G/C base content distribution statistics. To ensure the accuracy of subsequent analyses, the SeqPrep^2^ and Sickle^3^ software were used to control the quality of the original sequencing data, to obtain high-quality clean data. After quality control, statistics and quality evaluation were again performed on the clean data. We used the Trinity software^4^ to splice the sequencing results, and the TransRate software^5^ to optimize and filter the obtained initial assembly sequences. The Benchmarking Universal Single-Copy Orthologs software (BUSCO)^6^ utilizing single-copy orthologous genes was applied to assess the assembly integrity of the transcriptome. The open reading frame (ORF) coding frame of the transcript was identified, and then HMMER3 (v 3.1b2) mapping was performed. The assembled transcriptome sequences were annotated using six major databases (NR, Swiss-Prot, Pfam, COG, GO, and KEGG). Annotations with NR, Swiss-Prot, and COG were done using the Diamond software (v 0.8.37.99) with an *E*-value cut-off of 10^–5^. The GO annotations of unigenes were obtained using Blast2GO (v 2.5.0) software, and the KOBAS (v 2.1.1) software was used to provide KEGG functional classifications. The data were analyzed on the free online platform of Majorbio Cloud Platform^7^.

### Differential Expression Analysis

RSEM^8^ was used to compare the quality-controlled sequencing data with the assembled transcriptome sequences through the comparison software Bowtie. We estimated the expression abundance of genes and transcripts based on the comparison results. TPM (Transcripts Per Million reads) was used as an expression indicator. The DESeq2 software based on negative binomial distribution was used for statistical analysis of raw counts. We screened for differentially expressed genes (DEGs) between groups based on P_adjust less than < 0.05, and the absolute value of log_2_FC greater than 1. BH (FDR correction with Benjamini/Hochberg) was used for multiple test correction. DEGs between groups were analyzed by Venn to obtain the co-expressed and specific DEGs.

KEGG enrichment analysis was performed on each group of up- and down-regulated DEGs. We also conducted a KEGG enrichment analysis for continuous up-related DEGs throughout the overwintering. All DEGs were analyzed using STEM (Short Time-series Expression Miner)^9^ time-series expression trend analysis (Ernst and Bar-Joseph, 2006). SI_1 and SI_2, SI_3 and SI_4, SI_5 and SI_6, SI_7 and SI_8, SI_9 and SI_10 and SI_11 and SI_12 were combined into six orders. There were 30 time-series profiles. The significance level was *p* < 0.01. KEGG enrichment analysis was performed on DEGs in the Profile whose trends followed changes in cold resistance. The data were analyzed on the free online platform of Majorbio Cloud Platform (see text footnote 7).

### Quantitative RT-PCR (qRT-PCR) Analysis

We used qRT-PCR to verify the reliability of transcriptome sequencing. Gene expression levels were normalized to the β-actin housekeeping gene (Yang et al., 2019). We designed specific primers using the Primer3Plus web tool^10^, and primers were listed in Supplementary Table 1. qRT-PCR was conducted using the Bio-Rad CFX96 PCR System (Hercules, CA, United States) with SYBR Premix Ex Taq II (Takara, Dalian, China). Each PCR reaction was conducted in a 12.5-μL reaction mixture containing 6.25 μL of SYBR Premix Ex Taq II, 0.5 μL of each primer, 1 μL of cDNA template, and 4.25 μL of ddH_2_O. The amplification program was as follows: 95°C for 30 s; followed by 40 cycles of 95°C for 5 s, 60°C for 30 s, and 95°C for 10 s; then 65°C to 95°C in increments of 0.5°C for 5 s to generate the melting curves. Three biological replicates and three technical replicates were used for analysis. The relative expression of the genes was calculated according to the 2^–△^^△^^*CT*^ method (Livak and Schmittgen, 2001).

### Metabolite Extraction

The LC-MS parameters were referenced to the previous method (Yuan et al., 2012). Each larva was placed in a 1.5-ml micro-centrifuge tube and snap-freeze the tissue sample in liquid nitrogen (−196°C). The 80% (vol/vol) acetonitrile solution was prepared for metabolite extraction. 40 ml of HPLC-grade acetonitrile was added to a 50-ml polypropylene conical tube, followed by 10 ml of LC/MS-grade water solution. Add 150 μl of 80% (vol/vol) HPLC-grade acetonitrile (cooled to −80°C) to 35 milligrams of ground whole larva in a 1.5 ml microcentrifuge tube. Each sample was ground for 1–2 min with small pestle on dry ice in the tube, then vortex for 1 min at 4°C and incubate for 4 h at −80°C (Vortex-Genie2, Scientific Industries, United States). The sample was centrifuged at 14,000 g for 10 min using a refrigerated centrifuge at 4°C. Supernatant was transferred the to a new 1.5-ml microcentrifuge tube and stored at −80°C. Add 400 μl of 80% (vol/vol) acetonitrile (cooled to −80°C) to the precipitate. Vortex for 1 min at 4°C, and then incubate for 30 min at −80°C. Centrifuge at 14,000 g for 10 min at 4°C. Transfer and combine the supernatant from both extractions. The extraction was centrifuged again at 14,000 g for 10 min at 4°C. Transfer the supernatant to a new 1.5-ml microcentrifuge tube. SpeedVac to a pellet using no heat (Genevac miVac, Tegent Scientific Ltd.,England). The injection volume on the machine was 2 μL. All samples were extracted at LipidALL Technologies Company (Changzhou, China).

### Untargeted Metabolomics Instrumental Analysis

The ACQUITY UPLC HSS T3 1.8 μm, 2.1 × 100 mm columns (Waters, Dublin, Ireland) were adopted into the present study. Ultra-performance Liquid Chromatography (Agilent 1290 II, Agilent Technologies, Germany) coupled to Quadrupole-TOF MS (5600 Triple TOF Plus, AB SCIEX, Singapore) was applied to acquire metabolome data. The MS parameters for detection were: ESI source voltages were + 5.5 and -4.5 kV, respectively; vaporizer temperature, 500°C; drying gas (N2) pressure, 50 psi; nebulizer gas (N2) pressure, 50 psi; curtain gas (N2) pressure, 35 psi; The scan range was m/z 60–800. Information-dependent acquisition mode was used for MS/MS analyses of the metabolites. The collision energy was set at 35 ± 15 eV. Data acquisition and processing were performed using Analyst^®^ TF 1.7.1 Software (AB Sciex, Concord, ON, Canada). The internal standard includes phenylalanine D8, tryptophan D8, isoleucine D10, asparagine 13C4, methionine D3, valine D8, proline D7, alanine D4, glycine D2, serine D3, glutamate D5, aspartate D3, arginine D7, glutamine D5, lysine D9, histidine D5, and taurine D2. All samples were measured at LipidALL Technologies Company (Changzhou, China).

### Metabolomic Data Analysis

All detected ions were extracted using MarkerView 1.3 (AB Sciex, Concord, ON, Canada) into Excel in the format of two dimensional matrix, including mass to charge ratio (*m/z*), retention time, and peak areas, and isotopic peaks were filtered. PeakView 2.2 (AB Sciex, Concord, ON, Canada) was applied to extract MS/MS data, and perform comparison with Metabolites database (AB Sciex, Concord, ON, Canada), HMDB, METLIN, and standard references to annotate ion ID. Self-compiled R program LipidALL Technologies Company, Changzhou, China) was used for statistical analysis.

The analysis of metabolome data includes the identification of different metabolites and Metabolic Pathway Analysis (MetPA) (Xia and Wishart, 2010). We performed a principal component analysis on the data using R (4.0.2) (LipidALL Technologies Company, Changzhou, China). A cluster analysis was performed, and heat maps were drawn using R (4.0.2) (LipidALL Technologies Company, Changzhou, China). Orthogonal partial least squares discriminant analysis (OPLS-DA) and Mann-Whitney-U hypothesis tests were used to screen different metabolites using R (4.0.2) (LipidALL Technologies Company, Changzhou, China). We drew a volcano map of the difference between the groups using R (4.0.2) (LipidALL Technologies Company, Changzhou, China). The model species fruit fly (*D. melanogaster*) (KEGG) pathway database was used as reference database, with the species code dme. The database contains 81 metabolic pathways. This pathway analysis was done using a two-part method, involving a hypergeometric test (ORA) and relative betweenness centrality. The input metabolites were only those with *P* < 0.05 in the Mann-Whitney-U test, and the metabolites with KEGG IDs that did not match were removed. Up-regulated and down-regulated metabolites were judged based on fold change.

### Integrated Analysis of Transcriptomics and Metabolomics

To obtain the co-pathway, we compared the differential metabolic pathways obtained by enrichment analysis of the transcriptome and metabolome of each group of SI_1∼12. Pearson correlation analysis was performed on the log_2_FC of DEGs enriched in co-pathway and the fold change of metabolites enriched in co-pathway. A heat map was drawn based on the correlation coefficient using R (4.0.0). We screened for metabolites and differential genes with correlation coefficients =0.5 and =-0.5. We used Cytoscape (Cytoscape_v3.7.1) (Shannon et al., 2003) to draw a disordered co-expression network based on the correlation coefficients.

## Results

### RNA-Seq

**FIGURE 1:**
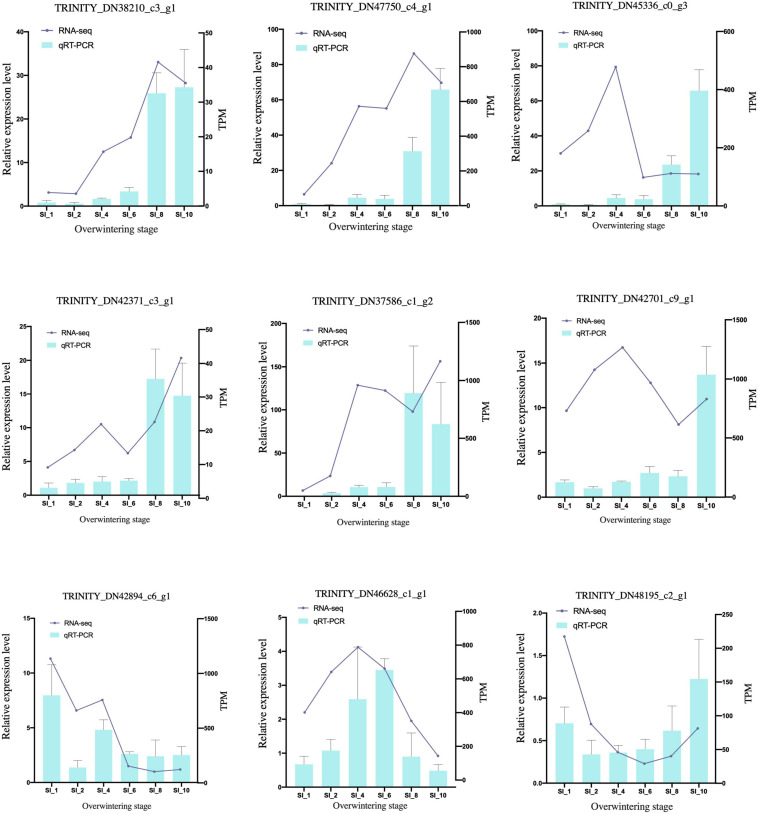
qRT-PCR validation of candidate genes. The qRT-PCR results (blue columns) were compared with RNA-seq data (purple lines). The relative expression level of qRT-PCR is shown on the y-axis to the left; and the normalized expression level (TPM) of RNA-seq is indicated on the y-axis to the right. SI_13 was set as control group.

After quality control, 2.08 GB of clean reads was obtained through transcriptome sequencing. The sequencing results of 39 samples showed no AT/GC separation, and the average GC content was 44.46%. The percentage of bases with Phred scores at the Q30 level (an error probability of 1%) of the sequencing results were all > 94.1%. Using Trinity to assemble the clean data, 293,093 transcripts and 195,129 unigenes were obtained. The BUSCO score was 96.7% (36.6%). The clean reads of each sample were mapped with the reference sequence assembled by Trinity, and the mapping results of each sample were obtained. The average mapping rate was 81.69%, and the mapping rate of each sample was > 79.45%. According to the qRT-PCR results (Figure 1 and Supplementary Table 1), the transcriptome sequencing result was reliable and universal. The datasets transcriptome for this study can be found in the NCBI SRA database under accession number PRJNA681480.

### Transcriptome Differential Expression Analysis

**FIGURE 2:**
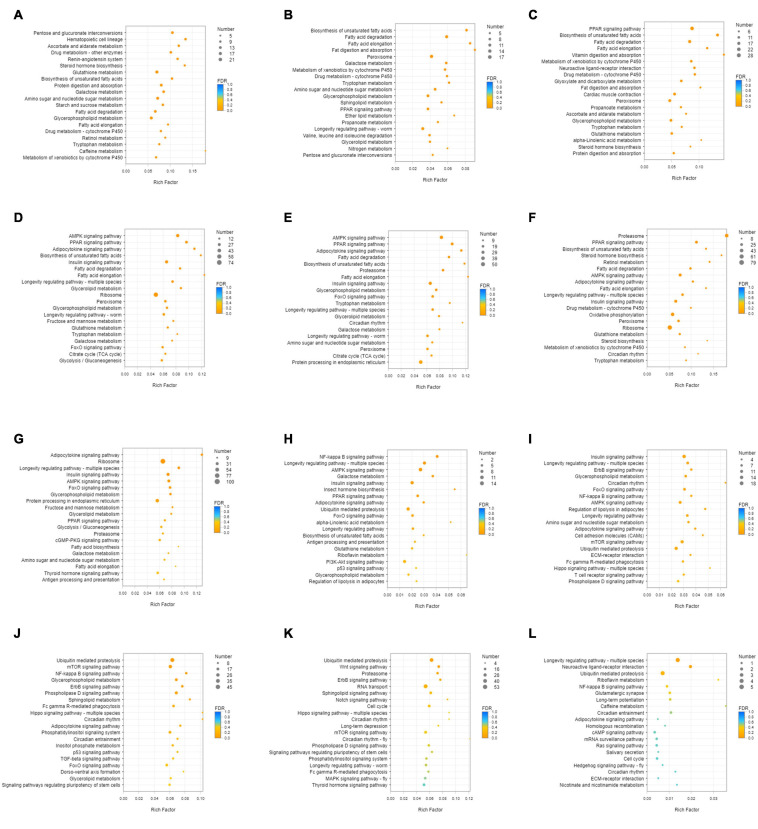
KEGG enrichment analysis of up-regulated DEGs. **(A)** DEGs of SI_1 **(B)** DEGs of SI_2 **(C)** DEGs of SI_3 **(D)** DEGs of SI_4 **(E)** DEGs of SI_5 **(F)** DEGs of SI_6 **(G)** DEGs of SI_7 **(H)** DEGs of SI_8 **(I)** DEGs of SI_9 **(J)** DEGs of SI_10 **(K)** DEGs of SI_11 **(L)** DEGs of SI_12. The picture shows the top 20 differential metabolic pathways with the smallest *P*-value.

**FIGURE 3:**
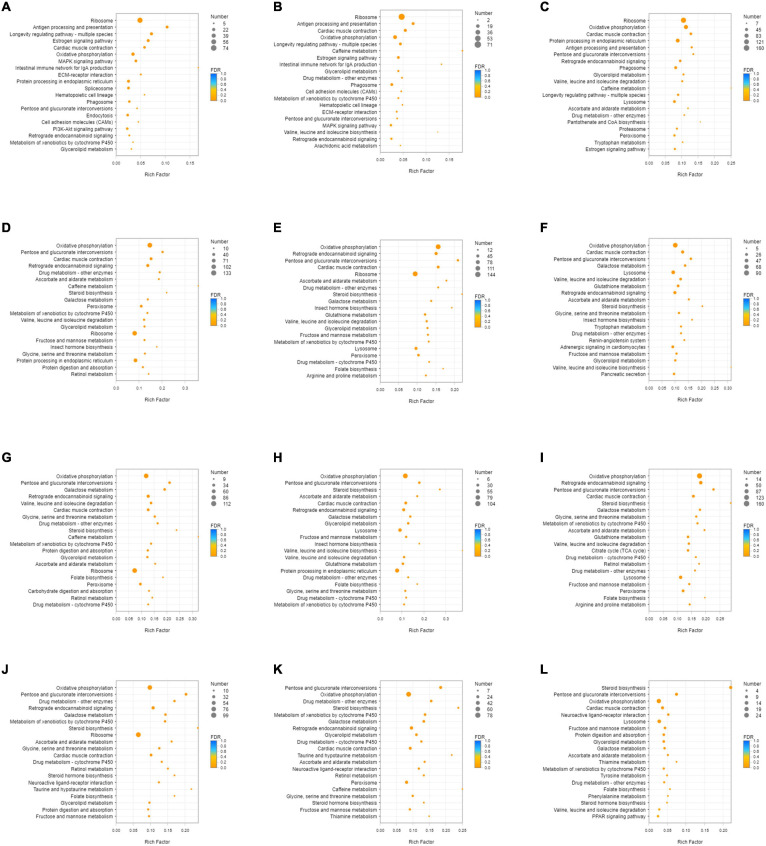
KEGG enrichment analysis of down-regulated DEGs. **(A)** DEGs of SI_1 **(B)** DEGs of SI_2 **(C)** DEGs of SI_3 **(D)** DEGs of SI_4 **(E)** DEGs of SI_5 **(F)** DEGs of SI_6 **(G)** DEGs of SI_7 **(H)** DEGs of SI_8 **(I)** DEGs of SI_9 **(J)** DEGs of SI_10 **(K)** DEGs of SI_11 **(L)** DEGs of SI_12. The picture shows the top 20 differential metabolic pathways with the smallest *P*-value.

**FIGURE 4:**
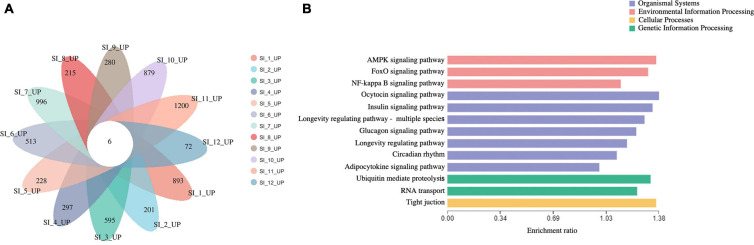
**(A)** venn analysis of up-regulated DEGs of each group. **(B)** KEGG enrichment analysis of continuously 6 up-regulated DEGs.

Unigenes with significant differences in expression (with *P* < 0.05) and an absolute value of up/down fold change ≥ 1 were defined as DEGs. The number of DEGs in each group is shown in Table 1. The DEGs of each group were enriched in the KEGG database (*P* < 0.05), and the two categories of human diseases and drug development were excluded. The enrichment of up- and down-regulated DGEs is shown in Supplementary Table 2. Top 20 metabolic pathways with the smallest *p*-value are shown in Figures 2, 3. A Venn analysis was performed on DEGs in each group. Six DEGs were differentially up-regulated in the 12 groups, and 39 DEGs were differentially down-regulated in the 12 groups. KEGG enrichment analysis was performed on the up-regulated DEGs, and 13 metabolic pathways were enriched (Figure 4 and Supplementary Table 2), including “Oxytocin signaling pathway” (map04921), “AMPK signaling pathway” (map04152), “Tight junction” (map04530), “Insulin signaling pathway” (map04910), “Ubiquitin mediated proteolysis” (map04120), “FoxO signaling pathway” (map04068), “Longevity regulating pathway—multiple species” (map04213), “RNA transport” (map03013), “Glucagon signaling pathway” (map04922), “Longevity regulating pathway” (map04211), “NF-kappa B signaling pathway” (map04064), “Circadian rhythm” (map04710), and “Adipocytokine signaling pathway” (map04920).

**TABLE 1:** Statistics of the number of DEGs.

Group	Up-related	Down-related	Total
SI_1	1,806	1,454	3,260
SI_2	1,356	1,107	2,463
SI_3	2,183	3,642	5,825
SI_4	3,057	4,685	7,742
SI_5	3,378	4,958	8,336
SI_6	3,165	3,862	7,027
SI_7	4,821	5,267	10,088
SI_8	1,776	3,880	5,656
SI_9	2,600	5,876	8,476
SI_10	4,187	5,008	9,195
SI_11	4,108	4,833	8,941
SI_12	468	1,171	1,639

### Time Series Expression Analysis of DEGs

**FIGURE 5:**
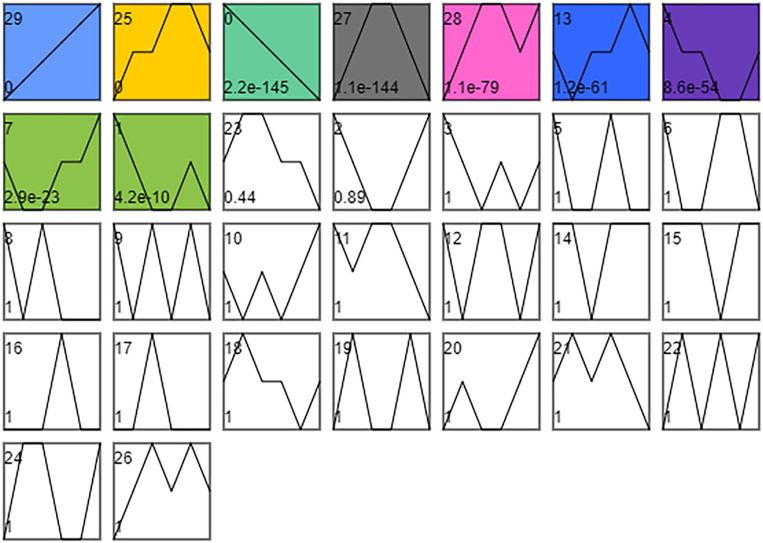
STEM timing analysis of DEGs. Combine SI_1 and SI_2, SI_3 and SI_4, SI_5, and SI_6, SI_7 and SI_8, SI_9, and SI_10, SI_11, and SI_12 into 6 orders. The number of time series profiles is 30. The significance level is *p* < 0.01.

As shown in Figure 5, the time series analysis identified nine significantly different gene expression patterns (*p* < 0.01). KEGG enrichment analysis was performed on the DEGs in the three profiles whose expression patterns related to the changes in cold tolerance, including Profile 25, Profile 27, and Profile 13.

We classified 1,783 DEGs into Profile 25. KEGG enrichment analysis was performed on these DEGs, and 15 differential metabolic pathways were enriched (*P*< 0.5; Supplementary Table 3). These pathways were the “P53 signaling pathway” (map04115), “Insulin signaling pathway” (map04910), “mTOR signaling pathway” (map04150), “Regulation of lipolysis in adipocytes” (map04923), “Longevity regulating pathway” (map04211), “PI3K-Akt signaling pathway” (map04151), “D-Glutamine and D-glutamate metabolism” (map00471), “Caffeine metabolism” (map00232), “Glycerophospholipid metabolism” (map00564), “Aldosterone-regulated sodium reabsorption” (map04960), “Longevity regulating pathway-worm” (map04212), “ECM-receptor interaction” (map04512), “FoxO signaling pathway” (map04068), “AMPK signaling pathway” (map04152), and “NF-kappa B signaling pathway” (map04064).

We classified 1,110 DEGs into Profile 27. KEGG enrichment analysis was performed on these DEGs, and ten differential metabolic pathways were enriched (*P* < 0.5; Supplementary Table 4). These pathways were the “Pentose and glucuronate interconversions” (map00040), “FoxO signaling pathway” (map04068), “Galactose metabolism” (map00052), “Fructose and mannose metabolism” (map00051), “Longevity regulating pathway—multiple species” (map04213), “Lysosome” (map04142), “Glycerolipid metabolism” (map00561), “Phenylalanine, Tyrosine and tryptophan biosynthesis” (map00400), “alpha-Linolenic acid metabolism” (map00592), “Sphingolipid metabolism” (map00600), and “Neuroactive ligand-receptor interaction” (map04080). We classified 1,120 DEGs into Profile 13. KEGG enrichment analysis was performed on these DEGs, but no differential metabolic pathway was enriched (*P* < 0.5).

### Differential Metabolite Identification and Differential Metabolic Pathway Enrichment Analysis

**FIGURE 6:**
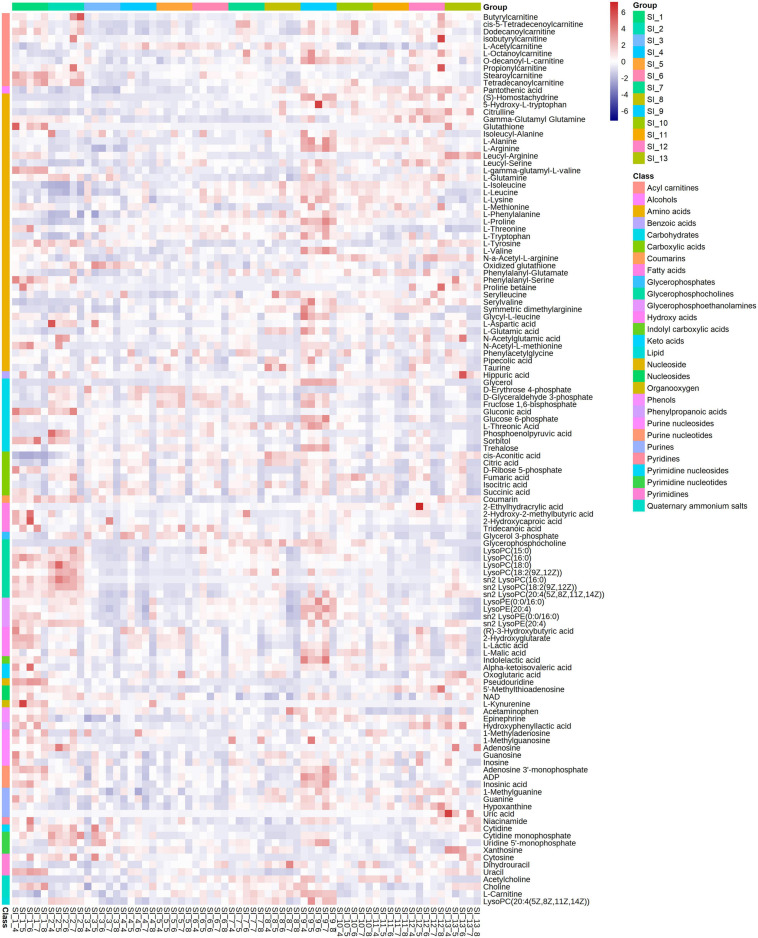
Correlation analysis of all identified differential metabolites from SI_1∼SI_13 to draw a heat map. 122 metabolites are divided into 28 clusters. The significance level is *p* < 0.05.

The metabolome included 122 metabolites belonging to 28 classes, including amino acids, acylcarnitines, and carbohydrates (Figure 6). Among these, amino acids were the class with the most metabolites (*n* = 38). The glycerophosphocholines (GPCs) content was the highest among the metabolites (Supplementary Figure 2).

**FIGURE 7:**
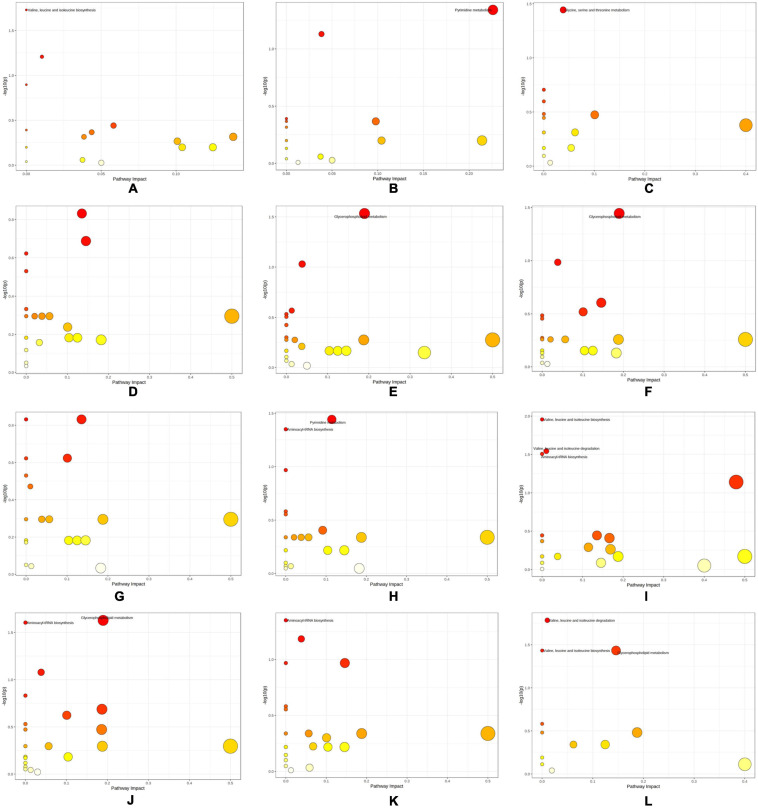
Differential metabolic pathways obtained by KEGG enrichment analysis of differential metabolites. **(A)** SI_1 **(B)** SI_2 **(C)** SI_3 **(D)** SI_4 **(E)** SI_5 **(F)** SI_6 **(G)** SI_7 **(H)** SI_8 **(I)** SI_9 **(J)** SI_10 **(K)** SI_11 **(L)** SI_12.

The differential metabolites of each group and enriched metabolic pathways according to MetPA are shown in Figure 7 and Supplementary Table 5. “Pyrimidine metabolism” (map00240), “Valine, leucine, and isoleucine degradation” (map00280), “Valine, leucine, and isoleucine biosynthesis” (map00292), “Glycine, serine, and threonine metabolism” (map00260), “Aminoacyl-tRNA biosynthesis” (map00970), and “Glycerophospholipid metabolism” (map00564) were the pathways with significant differences in the enrichment of metabolic pathways in each group.

### Interaction Analysis of Metabolomics and Transcriptomics

We compared the pathway enrichment results of the metabolomics and transcriptomics of each group (SI_1∼12) to identify significant differences (*P* < 0.05). There were four co-pathway enrichment results of the two omics approaches: “Valine, leucine, and isoleucine degradation”; “Valine, leucine, and isoleucine biosynthesis”; “Glycine, serine, and threonine metabolism”; and “Glycerophospholipid metabolism.” There were 80 DEGs and nine differential metabolites enriched in the glycerophospholipids metabolism pathway, which was the largest number of the four co-pathways.

**FIGURE 8:**
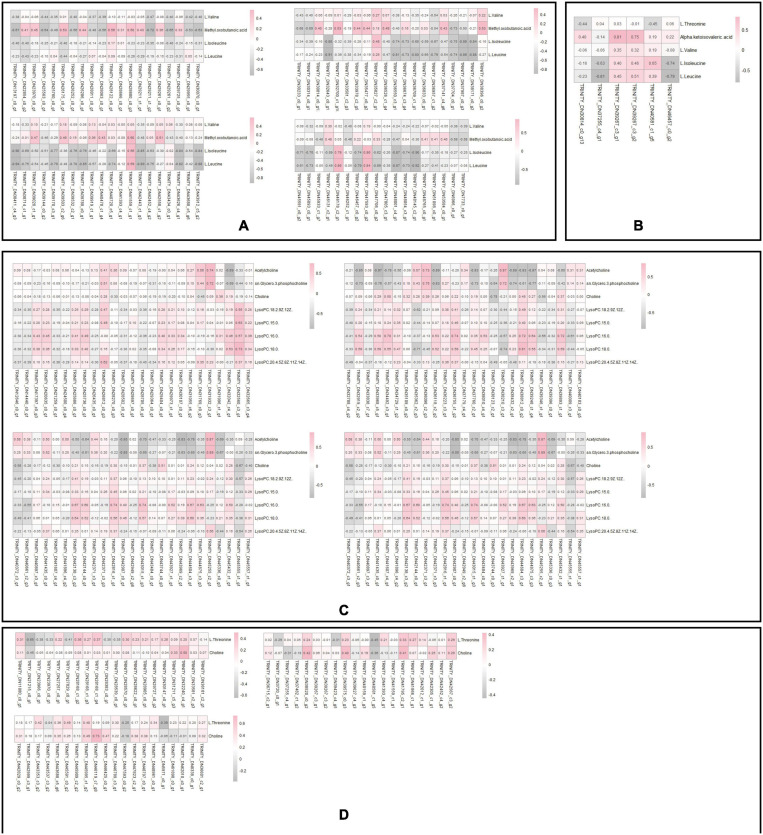
**(A)** Correlation analysis between the differential metabolites and DEGs of Valine, leucine and isoleucine degradation. Pink indicates positive correlation, and gray indicates negative correlation. **(B)** Correlation analysis between the differential metabolites and DEGs of Valine, leucine and isoleucine biosynthesis. Pink indicates positive correlation, and gray indicates negative correlation. **(C)** Correlation analysis between the differential metabolites and DEGs of Glycerophospholipid metabolism. Pink indicates positive correlation, and gray indicates negative correlation. **(D)** Correlation analysis between the differential metabolites and DEGs of Glycine, serine and threonine metabolism. Pink indicates positive correlation, and gray indicates negative correlation.

**FIGURE 9:**
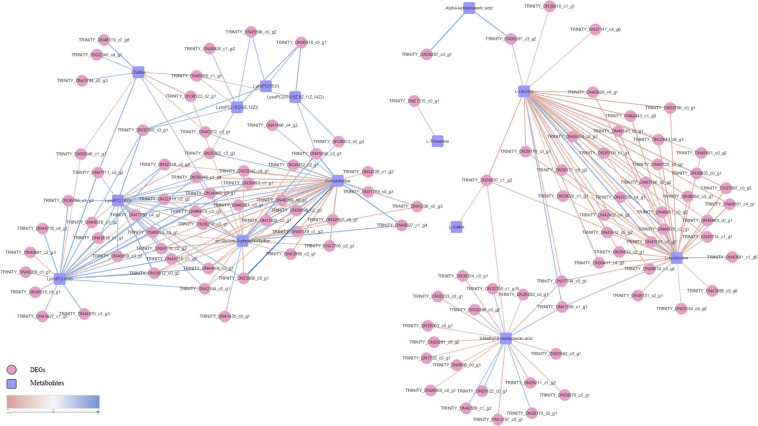
*S. insularis* metabolome and transcriptome Co-pathway related DEGs and metabolites interaction network. The pink circles represent DEGs. The blue squares represent differential metabolites. The color of the line represents correlation, red represents negative correlation, and blue represents positive correlation.

Pearson correlation analysis was performed on differential metabolites (fold change) and DEGs (Log_2_FC) enriched in each metabolic pathway and a heat map was drawn (Figure 8). We used differential metabolites and DEGs whose correlation coefficient *R* ≥ 0.5 in all metabolic pathways to draw a disordered interaction network diagram (Figure 9). We selected 26 genes where there may be an interaction with at least three metabolites: TRINITY_DN34492_c3_g1, TRINITY_DN46674_c3_g3, TRINITY_DN45833_c3_g1, TRINITY_DN44604_c3_g1, TRINITY_DN43015_c1_g3, TRINITY_DN42144_c0_g1, TRINITY_DN39248_c1_g4, TRINITY_DN38812_c0_g2, TRINITY_DN38210_c3_g1, TRINITY_DN36957_c1_g2, TRINITY_DN36118_c2_g2, TRINITY_DN32919_c2_g2, TRINITY_DN32560_c3_g1, TRINITY_DN56418_c0_g1, TRINITY_DN48061_c3_g1, TRINITY_DN47750_c4_g1, TRINITY_DN47711_c3_g2, TRINITY_DN45555_c1_g1, TRINITY_DN45255_c2_g1, TRINITY_DN42138_c3_g2, TRINITY_DN41559_c1_g1, TRINITY_DN39893_c1_g1, TRINITY_DN37704_c0_g1, TRINITY_DN35362_c2_g3, TRINITY_DN33968_c5_g1, and TRINITY_DN32042_c4_g1. We also screened out seven metabolites that might interact with at least 20 DEGs, including L-Isoleucine, L-Leucine, Acetylcholine (AcChol), sn-Glycero-3-phosphocholine, LysoPC (16:0), LysoPC (18:0) and 3-Methyl-2-oxobutanoic acid.

**FIGURE 10:**
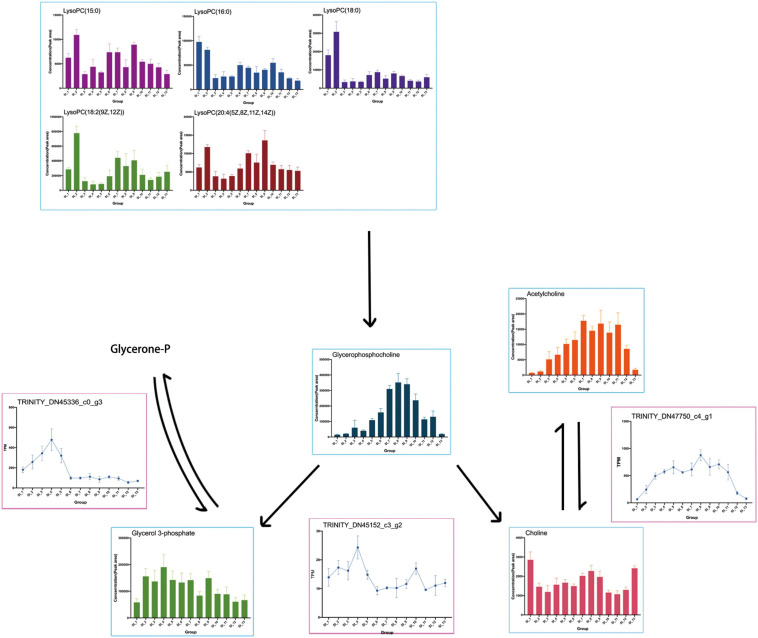
Presumed glycerophospholipid metabolism in the *S. insularis* larvae during the overwintering. The figure shows the content of metabolites in the pathway and the expression of DEGs that may be related to enzymes.

The results of the omics interaction analysis showed that the KEGG pathway “glycerophospholipid metabolism” might be a key pathway in the overwintering period of *S. insularis*. According to the metabolome results, the changing trend of glycerophosphocholine throughout the overwintering period was first a significant increase, followed by a decrease (Figure 10), which was the same as the changing trend of the cold tolerance of *S. insularis* larvae. The changing trend of choline during the wintering period also increased first and then decreased (Figure 10). Glycerol 3-phosphate (G3P) fluctuated upward in the early overwintering period, but gradually decreased in the middle and post overwintering period (Figure 10). At the same time, we noticed differences in the content of LysoPC (15:0), LysoPC (16:0), LysoPC (18:0), LysoPC [18:2 (9Z,12Z)], and LysoPC [20:4 (5Z,8Z,11Z,14Z)] (Figure 10). Moreover, our metabolomics results had significant changes in the content of LPEs and sn-LPCs which were not enriched in “Glycerophospholipid metabolism” pathway (Supplementary Figure 3). The concentrations of sn2-LysoPC (16:0) and sn2-LysoPC [18:2 (9Z,12Z)] were significantly higher before overwintering. The concentrations of LysoPE (0:0/16:0) and LysoPE (20:4) increased significantly when the temperature was the lowest during the overwintering. Sn2-LysoPC [18:2(9Z,12Z)]’s concentrations were higher than other GPCs and GPEs.

Transcriptome results showed the DEG annotated to the glycerophosphocholine phosphodiesterase GPCPD1 in the “glycerophospholipid metabolism” pathway: TRINITY_DN45152_c3_g2, which tended to first increase and then decrease while rise again at SI_6 to SI_10 with another peak and then fall. TRINITY_DN47750_c4_g1 was annotated as responsible for the expression of the acetylcholinesterase, which catalyzes the synthesis of Choline (Chol) from AcChol. This DEG was highly expressed when the cold tolerance of the *S. insularis* larvae peaked. We found that TRINITY_DN45336_c0_g3 was annotated on glycerol-3-phosphate dehydrogenase (NAD +), and its expression in SI_1 ∼ SI_6 had a tendency to increase first and then decrease. We also found some DEGs such as: TRINITY_DN37760_c2_g1, TRINITY_DN38210_c3_g1, TRINITY_DN38432_c2_g1, TRINITY_DN42144_c0_g1, TRINITY_DN45336_c0_g3, and TRINITY_DN42371_c3_g1, which have been annotated in “glycerophospholipid metabolism” pathway.

## Discussion

### Metabolic Pathways Are Associated With Continuous Up-Regulated DEGs, Which Might Maintain the Survival, Growth, and Development of *S. insularis* Larvae During the Overwintering Period

The low-temperature conditions in winter threaten the survival of *S. insularis* larvae. Therefore, during this period, the priorities become maintaining the most basic physiological activities. The enrichment analysis results of continuously up-regulated DEGs seemed to confirm our conjecture.

Ubiquitin-mediated proteolysis plays an important role in a wide range of basic cellular processes. These include regulating the cell cycle, regulating immune and inflammatory responses, controlling signal transduction pathways, and development and differentiation (Ciechanover et al., 2000). Relatively, it was speculated that the larvae of *S. insularis* might still overcome the harsh environment to maintain slow growth and development during the overwintering period. RNA transport is an important aspect of gene expression (Rodriguez et al., 2004). During the overwintering period, *S. insularis* larvae up-regulate DEGs related to cold tolerance, growth, and development, which is likely to require the expression of related genes in the RNA transport pathway. The transcription factors of the FoxO family regulate the expression of genes in cell physiological activities, including apoptosis, cell cycle control, glucose metabolism, anti-oxidative stress, and longevity (Eijkelenboom and Burgering, 2013). The nuclear factor kappa-B family regulates the expression of genes involved in various cellular processes, including inflammation, immunity, and cell survival (Karin and Lin, 2002). It was speculated that the role of the FoxO signaling pathway and NF-kB signaling pathway during the overwintering period was to ameliorate the damage caused by low temperature and maintain low-energy survival of the larvae of *S. insularis* during dormancy. Tight binding proteins are involved in maintaining barrier regulation, cell polarity, and gene transcription (Zihni et al., 2016). It has been speculated that the DEGs enriched in the tight-junctional pathway play a role in the growth and development process of *S. insularis*.

Longevity regulating pathway-multiple species were related to aging, which was used to resist a complex process of accumulation of molecular, cell, even organ damage leading to loss of function and increased vulnerability to disease and death (Bitto et al., 2015). It has been speculated that DEGs enriched in the longevity regulation pathway mainly resist low-temperature injury and maintain survival. Circadian rhythms help to synchronize the organism’s metabolic process with its environment, thereby improving biological adaptability. The circadian rhythm pathway can regulate active oxygen homeostasis and oxidative stress response, cell and body metabolism, DNA repair, and anti-aging signal pathways (Harmer et al., 2001). It was speculated that the DEGs enriched in the circadian rhythm also play a role in maintaining the survival and growth of the larvae of *S. insularis* during the overwintering period.

The continuous up-regulated DEGs of *S. insularis* larvae during the overwintering period were mostly involved in ensuring survival, growth, cell proliferation, and other physiological activities. Therefore, it was speculated that the larvae still ensure their slow growth and development under a low-temperature environment, indicating no diapause phenomenon during the overwintering period, which was consistent with the previous observation.

### Metabolic Pathways Related to Changes in Cold Tolerance in Time Series Analysis Might Play a Role in Improving the Cold Tolerance of *S. insularis*

According to the research on the cold tolerance of *S. insularis* larvae during the overwintering period, the cold tolerance first showed an increasing trend, then decreasing (Pei et al., 2020). In the time-series analysis results, the trend of Profile 25 and 27 also increased first and then decreased, similar to the change in cold tolerance. Therefore, it is speculated that the metabolic pathways enriched in DEGs in Profile 25 and 27 are related to cold tolerance.

Previous studies have reported that 5′-AMP-activated protein kinase (AMPK) plays an important role in regulating cell lipid and protein metabolism and a key role in the regulation of cell energy homeostasis. AMPK is the main regulator of catabolism and anabolic balance in cells (Hardie, 2007; Peers et al., 2007; Mihaylova and Shaw, 2011; Storey and Storey, 2012). The activation of AMPK signaling pathway is determined by the ratio of AMP and ATP (Gowans et al., 2013). In the fruit fly (*D. melanogaster*), AMPK functions as a regulator of energy homeostasis in cells and the whole body (Sinnett and Brenman, 2016). AMPK promotes the catabolic pathway that produces ATP and suspends the process of consuming ATP; AMPK also activates glucose and fatty acid uptake, glycolysis, and fatty acid oxidation (Hardie and Ashford, 2014). Under energy-limited conditions, AMPK inhibits fat production and promotes fatty acid oxidation (Hardie, 2011). AMPK activation also exerts inhibitory control on carbohydrate storage and protein synthesis. AMPK plays a role in insect cold tolerance. AMPK measurement in *Eurosta solidaginis* before the overwintering period (September) and during the overwintering period (February) showed that the AMPK activity of larvae in February was greatly increased by 70–90% (Rider et al., 2011).

Profile 25 had a trend similar to larvae cold tolerance according to our previous study (Pei et al., 2020). The reason we paid attention to profile25 was to try to find relevant information that affects the cold tolerance of *S. insularis* larvae in winter. In Profile 25, we noticed that 522 DEGs were enriched in the AMPK signaling pathway, and there were also continuously up-regulated DEGs enriched in this pathway. Therefore, it was speculated that the AMPK signaling pathway might play a key role in the overwintering period of *S. insularis* larvae and might change the larvae’s metabolic type during the overwintering period (from anabolic metabolism to catabolism) and consume stored functional substances. According to our previous research, glycogen content decreased when cold tolerance increased (Pei et al., 2020), and this process might be regulated by AMPK signaling pathway according to previous study (Hardie, 2007; Peers et al., 2007; Mihaylova and Shaw, 2011; Storey and Storey, 2012). The AMPK signaling pathway might also reduce the larvae’s overwintering metabolic level, to ensure that energy consumption was reduced under low temperature and low intake conditions according to previous study. At the same time, larvae still ensured a certain concentration of antifreeze protection agents (such as fatty acid) in dynamic balance to maintain body fluid concentration and participate in cold-tolerant physiological activities. According to our previous research, *S. insularis* larval glycogen and total lipid are not exhausted, and there is no significant difference in protein content in overwinter period (Pei et al., 2020). The ultimate goal is to ensure that the larvae can survive the winter.

In Profile 27, we noticed pathways such as “Pentose and glucuronate interconversions,” “Fructose and mannose metabolism,” “Galactose metabolism,” and “Glycerolipid metabolism.” Those pathways were related to the use of energy by organisms. We speculated that these metabolic pathways might be regulated by AMPK signaling pathway, and some catabolism might occur in *S. insularis* larvae. Simultaneously, the larvae also used these metabolic activities to ensure energy supply during overwintering.

### Co-pathway in Metabolomics and Transcriptomics Might Be the Key to Affecting the Cold Tolerance of *S. insularis*

During the overwintering, the extracellular fluid of freezing tolerant insects freezes while the intracellular fluid does not freeze (Sinclair, 1999). Therefore, maintaining the cell membrane might be the key to their overwintering period. Phospholipids are the main components of cell membranes, and different phospholipid compositions affect the thickness and fluidity of cell membranes (Renne and de Kroon, 2018). In our study, according to the results of metabolomics, a variety of Lyso-PCs (LPC) and Lyso-PEs (LPE) changed during the overwintering period and LysoPC[18:2(9Z,12Z)] and LysoPC[20:4(5Z,8Z,11Z,14Z)] had a similar tend with cold tolerance of *S. insularis* larvae. The content of GPC increased first and then decreased. The content of G3P accumulated before the lowest temperature. Except the higher levels of SI_1 and SI_13, the content of Chol also tended to increase first and then decrease. According to the results of transcriptomics, we found that the gene expression involved in the degradation of LPCs was extremely low, however genes involved in the synthesis of Chol and G3P were expressed in large quantities, and the change trend was similar to the change in cold tolerance. In addition, we considered that the synthesis and decomposition in glycerophospholipid metabolism pathway seemed to occur at the same time. In our results, a DEG annotated in the decomposition of GPC were also expressed during the overwintering, and GPC was decomposed into G3P and Chol under the catalysis of this enzyme. According to our analysis, the “glycerophospholipid metabolism” pathway was an important KEGG metabolic pathway throughout the overwintering period. This pathway might participate in the physiological activities of stabilizing cell membranes during this period (Tomcala et al., 2006). However, in the choice of column in metabolomics determination, we put more emphasis on the accurate chromatographic separation of metabolites with high polarity. We still need further research to understand how glycerophospholipid metabolism participate in cold tolerance. In order to have a more in-depth understanding of the glycerophospholipid metabolic pathways in the cold tolerance of *S. insularis* larvae, we will conduct detailed studies on the content of weakly polar metabolites such as GLs, PCs, and PEs.

## Conclusion

In conclusion, through transcriptomics and metabolomics analyses during the overwintering period, we conclude that the AMPK signaling pathway and glycerophospholipid metabolism are likely to play key roles in the overwintering physiological process of *S. insularis* larvae in the field. During winter, the AMPK signal pathway played a signal transduction role. After receiving cold stimulation, it adjusted the metabolism from anabolism to catabolism to ensure energy supply and survival. The glycerophospholipid metabolism pathway is important because it allows the larvae to ensure the stability of cell membrane. Through omics analysis, we explained the physiological process of the *S.insularis* larvae overwintering in the wild, which is helpful for further research on its cold tolerance mechanism. Moreover, our research may also help to understand the possibility of its diffusion under global temperature changes.

## Data Availability Statement

The datasets presented in this study can be found in online repositories. The names of the repository/repositories and accession number(s) can be found in the article/Supplementary Material.

## Author Contributions

SZ, LR, and JP conceived and designed research. JP and YX conducted experiments. JP analyzed data and wrote the manuscript. All authors read and approved the manuscript.

## Conflict of Interest

The authors declare that the research was conducted in the absence of any commercial or financial relationships that could be construed as a potential conflict of interest.

## Footnotes

1 http://hannonlab.cshl.edu/fastx_toolkit/

2 https://github.com/jstjohn/SeqPrep

3 https://github.com/najoshi/sickle

4 https://github.com/trinityrnaseq/trinityrnaseq

5 http://hibberdlab.com/transrate/

6 http://busco.ezlab.org

7 www.majorbio.com

8 http://deweylab.github.io/RSEM/

9 http://www.sb.cs.cmu.edu/stem/

10 http://www.primer3plus.com/cgi-bin/dev/primer3plus.cgi

## Supplementary Material

Supplementary Figure 1 Beijing temperature record of the sampling month. Beijing temperature record of September 2018 to March 2019 (http://data.cma.cn) was shown in line chart. The red line indicated the monthly average high temperature. The blue line indicated the monthly average low temperature.

Supplementary Figure 2 Summary of metabolites in each category in the metabolome. 28 classes of metabolites were shown in bar chat. 13 groups were indicated in different color.

Supplementary Figure 3 Heatmap of concentrations (peak area) of all glycerophosphates, glycerophosphocholines and glycerophosphoethanolamines.

Supplementary Table 1 Primers of qRT-PCR.

Supplementary Table 2 The enriched KEGG metabolic pathway of each group.

Supplementary Table 3 The enriched KEGG metabolic pathway of profile 25.

Supplementary Table 4 The enriched KEGG metabolic pathway of profile 27.

Supplementary Table 5 Differential metabolites in each group and all metabolic pathways enriched.
