# Supplementary figures and images for: Transcriptomic and Metabolomic Data Reveal the Key Metabolic Pathways Affecting Streltzoviella insularis (Staudinger) (Lepidoptera: Cossidae) Larvae During Overwintering
Source: Front Physiol. 2021 Jun 18;12:655059. doi: 10.3389/fphys.2021.655059 (PMC8250450; doi:10.3389/fphys.2021.655059)

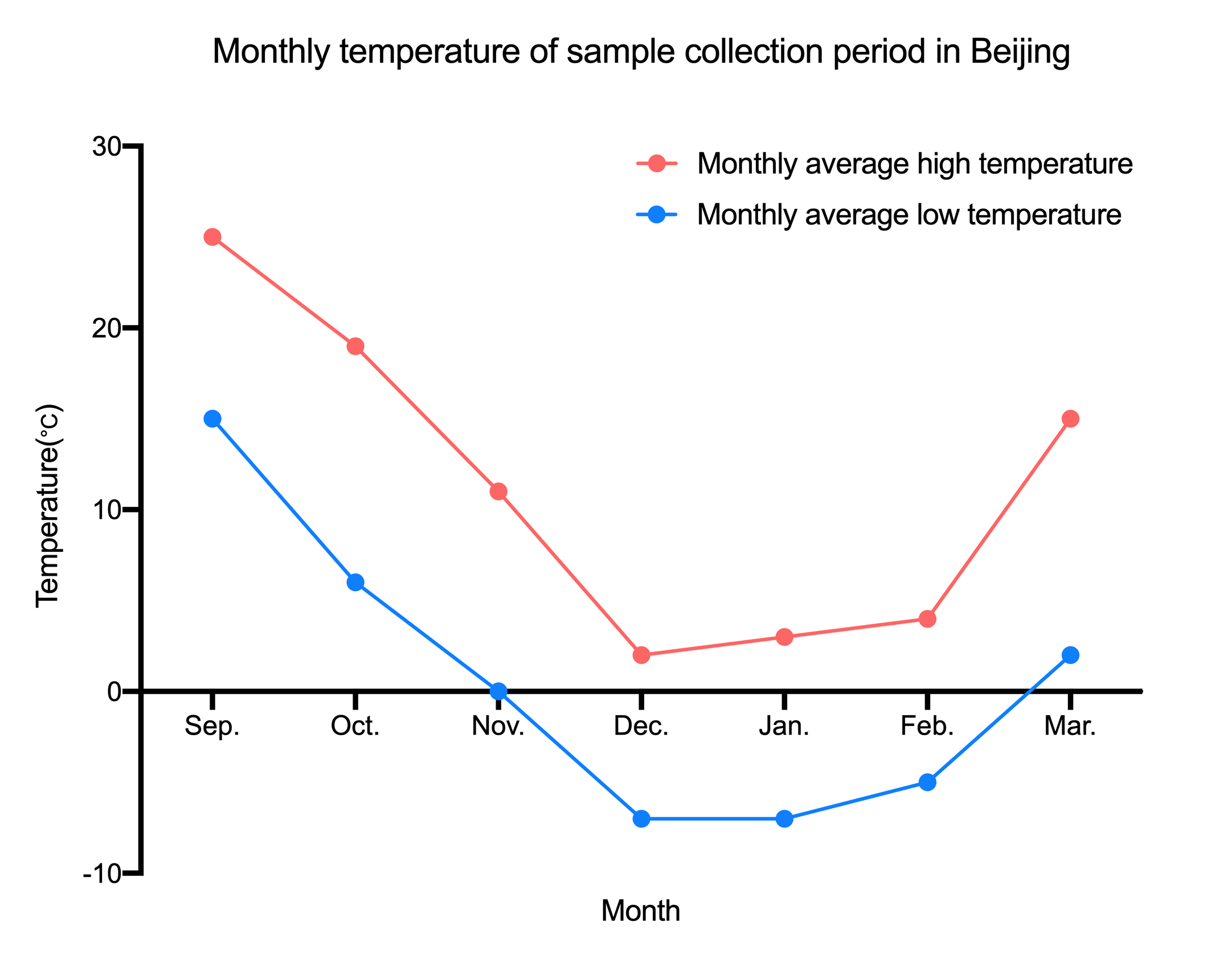

Supplement: Supplementary Figure 1 — Beijing temperature record of the sampling month. Beijing temperature record of September 2018 to March 2019 (http://data.cma.cn) was shown in line chart. The red line indicated the monthly average high temperature. The blue line indicated the monthly average low temperature. [file Image_1.TIFF]

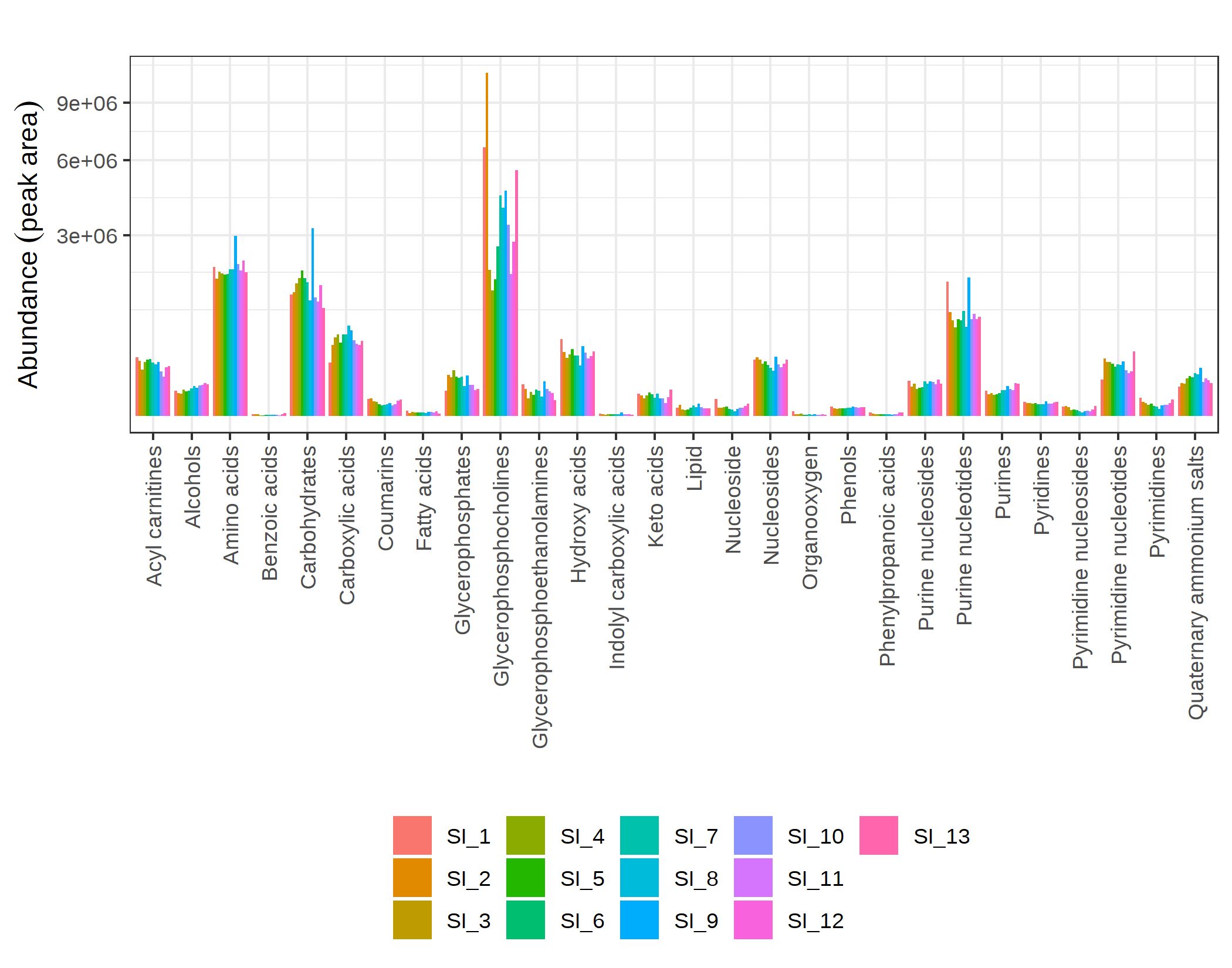

Supplement: Supplementary Figure 2 — Summary of metabolites in each category in the metabolome. 28 classes of metabolites were shown in bar chat. 13 groups were indicated in different color. [file Image_2.TIF]

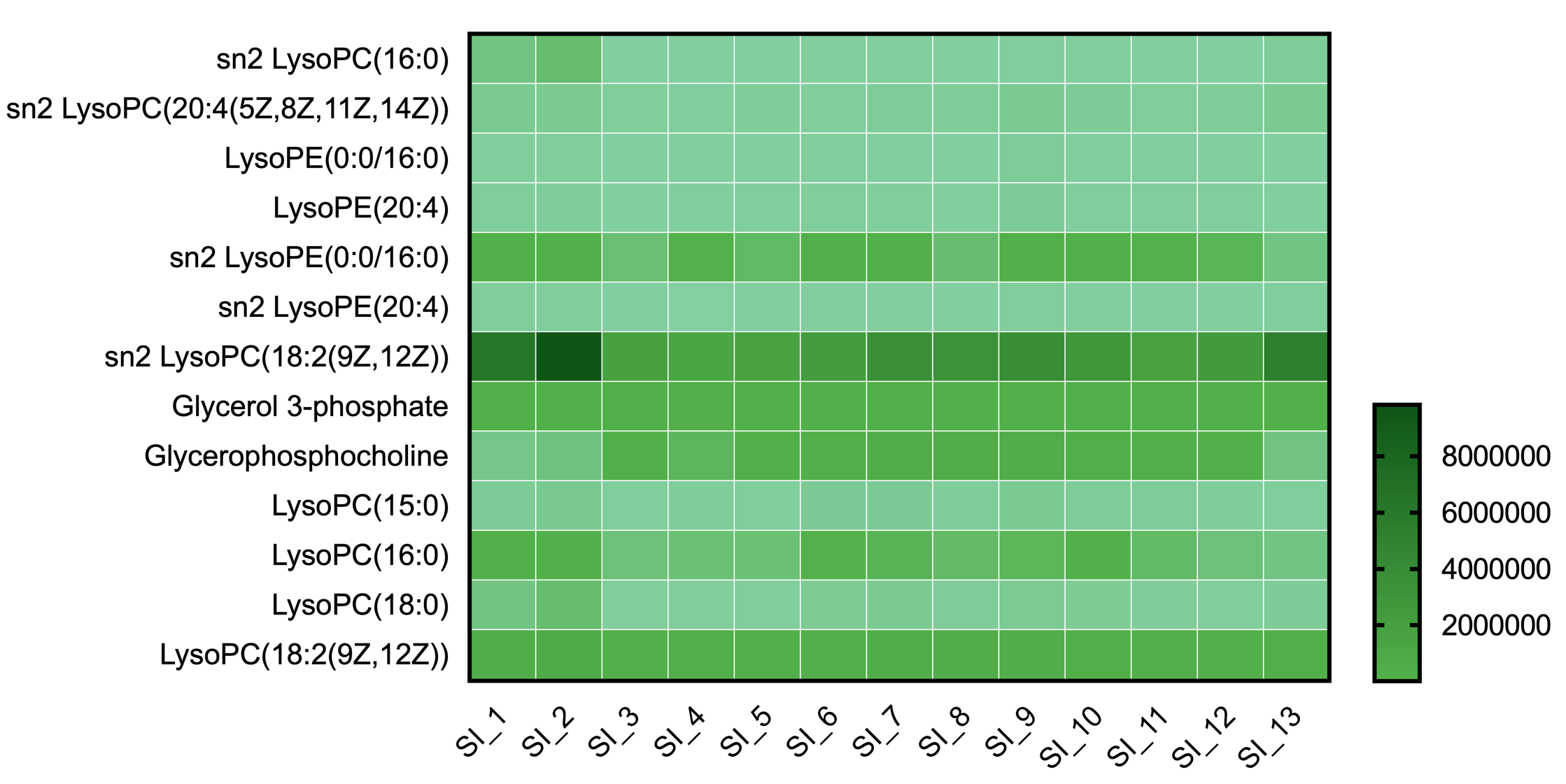

Supplement: Supplementary Figure 3 — Heatmap of concentrations (peak area) of all glycerophosphates, glycerophosphocholines and glycerophosphoethanolamines. [file Image_3.TIFF]
